# Editorial: Atrial fibrillation: selection of management strategy and evaluation of outcomes

**DOI:** 10.3389/fcvm.2024.1481893

**Published:** 2024-09-03

**Authors:** Daniel A. Gomes, Teresa Strisciuglio, Alexandre Almorad, Serge Boveda, Rui Providência

**Affiliations:** ^1^ Department of Cardiology, Hospital de Santa Cruz, Lisbon, Portugal; ^2^ Division of Cardiology, Department of Advanced Biomedical Sciences, Federico II University of Naples, Naples, Italy; ^3^ Heart Rhythm Management Centre, Postgraduate Program in Cardiac Electrophysiology and Pacing, Vrije Universiteit Brussel, Universitair Ziekenhuis Brussel, Brussels, Belgium; ^4^ Heart Rhythm Management Department, Clinique Pasteur, Toulouse, France; ^5^ Institute of Health Informatics Research, University College London, London, United Kingdom; ^6^ Barts Heart Centre, St Bartholomew’s Hospital, Barts Health NHS Trust, London, United Kingdom

**Keywords:** atrial fibrillation, arrhythmia, diagnosis, treatment, prognosis

**Editorial on the Research Topic**
Atrial fibrillation: selection of management strategy and evaluation of outcomes

Atrial fibrillation (AF) is the most common sustained arrhythmia worldwide and it is associated with significant healthcare burden from hospitalisations and complications (1). Focusing on improving cardiovascular outcomes and symptoms, contemporary AF management includes approaches for stroke prevention, rate or rhythm-control strategies, and the identification and treatment of associated cardiovascular risk factors and comorbidities (1). Catheter ablation, and particularly pulmonary vein isolation (PVI), is currently an established first-line treatment for symptomatic paroxysmal AF aiming for sinus rhythm maintenance (1). However, despite significant scientific and technological advances in the past decades, it is still associated with suboptimal outcomes in persistent AF (2). A tailored approach, by targeting patient-specific abnormal substrate and mechanisms of AF initiation and maintenance, is therefore necessary to improve success rates.

This Research Topic covered three active areas of contemporary AF research.

i. Prediction and improvement of outcomes in the new-onset AF population

A large UK linked primary and secondary care electronic health record (EHR) study including almost 200,000 patients with new-onset AF, showed that the most frequent healthcare interactions of patients with AF were cardiac, cerebrovascular, and peripheral-vascular when compared to non-AF age- and sex-matched controls. Using a novel methodology of EHR-wide association study, this work provides further data into the heterogeneity of AF presentation and associated comorbidities, before and after being diagnosed with arrhythmia, suggesting that the development of AF is a harbinger of worsening of cardiovascular and non-cardiovascular health. A better understanding of the leading causes of frequent healthcare services utilisation preceding the diagnosis of AF may help pinpoint high-risk subgroups that could benefit from intensive screening. This is particularly relevant if identifying such patients is followed by timely appropriate treatments, including stroke prevention, risk factor modification, and rhythm control, potentially improving prognosis. This hypothesis was corroborated by findings from: (i) a systematic review by Ma et al., including more than 95,000 patients with AF detected after stroke, which found that an early rhythm control strategy was associated with a 36% risk reduction in the incidence of recurrent cerebrovascular events when compared to usual care; (ii) a Korean nationwide cohort study with over 41,000 elderly patients with new-onset AF and followed for a median follow-up of 3.4 years. Combining early rhythm control and maintaining a healthy lifestyle was associated with the lowest risk of ischemic stroke and death in this patient group.

ii. Identification of patients less likely to respond to pulmonary vein isolation

It is long known that rhythm-control interventions are less effective in persistent AF forms (2). The presence of abnormal structural and electrical substrates outside the pulmonary veins and limitations in the achievement of persistent transmural lesions might contribute to this (1, 2). Creta and colleagues reported on the association between duration of surface ECG amplified P-wave (APW) in sinus rhythm and relapse following PVI for persistent AF. In this observational multicentre study including 295 patients undergoing cryoballoon PVI-only, an APW cut-off of >150 ms conferred a two-fold increase in risk of AF recurrence during a mean follow-up of 2.2 years. This simple measurement may help in differentiating patients who benefit most from a PVI-only approach vs. those where PVI-only is less likely to be successful, and where identification and treatment of further triggers might potentially be of benefit.

Hemodynamic changes and wall shear stress can also contribute to AF progression. Choi et al. showed that among 2,300 patients undergoing PVI, pulmonary artery pressure (PAP) ≥35 mmHg measured prior to the procedure through transthoracic echocardiography (TTE) was associated with AF recurrence. Importantly, this was true only for paroxysmal AF forms, possibly explained by the fact that hemodynamic abnormalities precede the development of structural modifications. Another factor to be taken into account is possible lack of precision when measurement of PAP through TTE in persistent AF patients (i.e., more likely to be in AF at the time of TTE).

iii. New mapping and ablation strategies

A pilot work from Squara et al. including 29 patients with persistent AF showed that high-density average complex interval (ACI) mapping on top of visual identification of fractionated electrograms may be useful for identification of active AF drivers, yielding a moderate discrimination (area under the curve 0.728) across 159 analysed atrial areas. If properly validated by larger-scale prospective studies, and if associated with better long-term arrhythmia-free survival, ACI mapping might be of interest for establishing a hierarchical approach by prioritizing ablation from the shortest to the longest ACI zone.

Aoyama and colleagues performed bi-atrial endocardial driver mapping using a real-time phase-mapping system and observed that baseline AF activation pattern mapping may aid in predicting freedom from arrhythmia, as driver activity levels were associated with higher recurrence rates following PVI. Future studies should assess whether adding substrate modification to PVI in these patients is associated to a better outcome.

Finally, Zhang et al. performed a systematic review on the efficacy and safety of third and fourth generation cryoballoons for PVI. Among 3,281 AF patients from thirteen observational studies, third and fourth generation cryoballoons seemed to be more effective and faster than the second generation cryoballoon, allowing documentation of real-time PVI in more patients, with a similar safety profile.

Further studies are necessary to clarify the potential impact of novel technologies and sources of energy (e.g*.*, ultra-low temperature cryoablation, pulsed-field ablation) in improving clinical outcomes in persistent AF.

Overall, this Research Topic covers several studies on AF management strategies and outcomes, offering insight into the challenges of improving patient care. To address these issues, future studies should focus on deeper understanding the biological and pathophysiological mechanisms of AF progression and maintenance. Ideally, the future of AF treatment should shift towards a more personalised, patient-tailored approach.
